# Identification of a potential diagnostic signature for postmenopausal osteoporosis *via* transcriptome analysis

**DOI:** 10.3389/fphar.2022.944735

**Published:** 2022-08-29

**Authors:** Rui Zeng, Tian-Cheng Ke, Mao-Ta Ou, Li-Liang Duan, Yi Li, Zhi-Jing Chen, Zhi-Bin Xing, Xiao-Chen Fu, Cheng-Yu Huang, Jing Wang

**Affiliations:** ^1^ Department of Physiology, School of Medicine, Jinan University, Guangzhou, China; ^2^ Department of Orthopedics, The First Affiliated Hospital of Jinan University, Guangzhou, China; ^3^ Department of Radiology, The First Affiliated Hospital of Jinan University, Guangzhou, China; ^4^ Department of Plastic Surgery, The First Affiliated Hospital of Jinan University, Guangzhou, China

**Keywords:** postmenopausal osteoporosis (PMOP), biomarkers, diagnostic signature, WGCNA, network pharmacology

## Abstract

**Purpose:** We aimed to establish the transcriptome diagnostic signature of postmenopausal osteoporosis (PMOP) to identify diagnostic biomarkers and score patient risk to prevent and treat PMOP.

**Methods:** Peripheral blood mononuclear cell (PBMC) expression data from PMOP patients were retrieved from the Gene Expression Omnibus (GEO) database. Differentially expressed genes (DEGs) were screened using the “limma” package. The “WGCNA” package was used for a weighted gene co-expression network analysis to identify the gene modules associated with bone mineral density (BMD). Least absolute shrinkage and selection operator (LASSO) regression was used to construct a diagnostic signature, and its predictive ability was verified in the discovery cohort. The diagnostic values of potential biomarkers were evaluated by receiver operating characteristic curve (ROC) and coefficient analysis. Network pharmacology was used to predict the candidate therapeutic molecules. PBMCs from 14 postmenopausal women with normal BMD and 14 with low BMD were collected, and RNA was extracted for RT-qPCR validation.

**Results:** We screened 2420 differentially expressed genes (DEGs) from the pilot cohort, and WGCNA showed that the blue module was most closely related to BMD. Based on the genes in the blue module, we constructed a diagnostic signature with 15 genes, and its ability to predict the risk of osteoporosis was verified in the discovery cohort. RT-qPCR verified the expression of potential biomarkers and showed a strong correlation with BMD. The functional annotation results of the DEGs showed that the diagnostic signature might affect the occurrence and development of PMOP through multiple biological pathways. In addition, 5 candidate molecules related to diagnostic signatures were screened out.

**Conclusion:** Our diagnostic signature can effectively predict the risk of PMOP, with potential application for clinical decisions and drug candidate selection.

## Introduction

Osteoporosis is the most common systemic bone disease in postmenopausal women (PMOP). This condition is characterized by decreased bone mineral density (BMD) and destruction of the bone tissue microstructure, resulting in decreased bone strength and increased fracture risk (Yao et al., 2019). The latest data show that the prevalence of osteoporosis in women over 50 years old in China is 29.1%, and the total number of patients is approximately 49 million. With the increase in the aging of society, by 2050, there will be 5.99 million cases of osteoporosis-related fractures in China every year (Cheng et al., 2021). Hip fracture is a common osteoporotic fracture in older postmenopausal women. Approximately 33% of women aged 90 years will experience a hip fracture, and these patients are older and have multiple comorbidities, leading to a poor prognosis (Ensrud et al., 2019). Osteoporosis has become a major public health problem worldwide, placing a heavy financial burden on patients and healthcare systems. Osteoporosis includes primary and secondary osteoporosis. The occurrence of primary osteoporosis is related to estrogen deficiency in women, decreased testosterone levels in men, and changes in hormone levels such as parathyroid hormone and calcitonin. Secondary osteoporosis is mainly caused by endocrine and metabolic diseases, connective tissue diseases, kidney diseases, digestive tract diseases, and drugs.

Most patients have no apparent symptoms in the early stage of osteoporosis, and the main clinical manifestations include pain, reduced height, limited activities, stooped posture, and respiratory system involvement. Most people lack awareness of osteoporosis and fail to detect the symptoms. Even when a brittle fracture occurs, there is no clear history of trauma or only a slight history of trauma (Glaser and Kaplan, 1976). Osteoporosis can be characterized by sparse trabecular bone and decreased BMD on imaging, but these imaging manifestations are affected by subjective factors and are not sensitive to early bone loss. Dual-energy X-ray absorptiometry (DXA) testing of BMD is strongly recommended by the World Health Organization (WHO). However, DXA cannot detect early bone loss, and studies of many clinical cases have suggested that DXA does not accurately assess the severity of osteoporosis and the risk of fracture. For example, in one report, the individuals who had the highest risk of future fractures among those who had bone density tests were rarely diagnosed with osteoporosis (T < -2.5) but were often diagnosed with reduced bone mass (−2.5 < T < -1) (Siris et al., 2014).

Therefore, transcriptome analysis may be helpful for the early diagnosis and prevention of osteoporosis. Based on the Gene Expression Omnibus (GEO) database, we used WGCNA to identify the top gene modules related to osteoporosis and conducted least absolute shrinkage and selection operator (LASSO) regression to build a diagnostic signature composed of 15 genes. The two most representative genes, *METTL4* and *RAB2A*, were subjected to RT–qPCR to verify the correlation between their expression and BMD. In addition, we explored the potential molecular mechanism of osteoporosis, which will contribute to the early diagnosis, treatment, and prevention of this disease. To discover novel osteoporosis drugs from our research, we explored the molecular targets of the diagnostic signature through network pharmacology.

## Materials and methods

### Microarray data collection and processing

The datasets were downloaded from the GEO database (https://www.ncbi.nlm.nih.gov/geo/) (Barrett et al., 2013), and the pilot cohort and discovery cohort were analyzed on the Affymetrix Human Genome U133A Array platform. As the pilot cohort, GSE56815 included 80 Caucasian females, 40 of whom had high BMD and 40 of whom had low BMD. In addition, we selected 20 postmenopausal subjects from the GSE13850 dataset as a discovery cohort: 10 women with high BMD and 10 women with low BMD. Raw data were read through the “affy” package (Gautier et al., 2004), and the RMA algorithm was used for background correction and data normalization. To verify the purity of peripheral blood mononuclear cells (PBMCs) in the pilot cohort, we used the “Cibersort” package.

### Identification of differentially expressed genes (DEGs)

Patients are classified into high- and low-BMD groups based on clinical information provided by uploaders in the pilot cohort. The “limma” package (Ritchie et al., 2015) was used to compare samples from the high- and low-BMD groups. Genes with adjusted *p*-value < 0.05 were defined as DEGs, DEGs with log2FC > 0 were defined as up-regulated DEGs, while those with log2FC < 0 were down-regulated DEGs. The “ggplot2” package (Ito and Murphy, 2013) was used to visualize the DEGs.

### Weighted gene coexpression network analysis

WGCNA is an algorithm to mine module information from high-throughput data. In this method, the module is defined as genes with similar expression trends. If these genes always have similar expression changes in a physiological or pathological process, it is reasonable to believe that they are functionally related and can be defined as a module. In this study, the “WGCNA” package (Langfelder and Horvath, 2008) was used to construct the weighted adjacency matrix by selecting appropriate thresholds, and the weighted adjacency matrix was transformed into a topological overlap matrix (TOM). The hierarchical clustering method was used to cluster the TOM matrix, and the dynamic tree-cutting algorithm was adopted to divide modules, each corresponding to a color, merge similar modules to find the module with the highest correlation with BMD, and extract the most significant genes associated with BMD in the module.

### Construction and validation of a genetic diagnostic signature of osteoporosis

LASSO logistic regression was used to reduce the dimensionality of genes in the BMD association module to construct a genetic diagnostic signature, which was generated by using the “glmnet” package (Friedman et al., 2010). Then, the signature was used to calculate the risk score of each patient. The corresponding coefficient of the gene weighted the expression values of these genes in each patient, and then, the weighted expression values were added to obtain the risk score of the patient, which was calculated as follows:

$$\mathrm{Risk}\;\mathrm{score}=\sum_{i-1}^{n}Exp_{i}*Coef_{i}$$

where n is the number of genes included in the signature, $Exp_{i}$ is the expression value of this gene of the patient, and $Coef_{i}$ is the coefficient of this gene in the signature. Finally, patients were classified into a high-risk group or a low-risk group according to the median value of the risk score.

Next, the “pROC” package (Robin et al., 2011) was used to draw the receiver operating characteristic curve (ROC) and determine the area under the curve (AUC). If the AUC was >0.8, the diagnostic effect was defined as good. In addition, we obtained another independent dataset, GSE13850, as the discovery cohort, applied the genetic diagnostic signature to the discovery cohort and defined its diagnostic effect in the discovery cohort according to the AUC.

### Functional annotation analysis

For determination of the potential biological pathways of osteoporosis, “clusterProfiler” package (Yu et al., 2012) was used to perform Kyoto Encyclopedia of Genes (KEGG), Gene Ontology (GO), Disease Ontology (DO) on the up- and down-regulated DEGs, the results for which adjust *p*-value<0.05 were considered statistically significant. Gene Set Enrichment Analysis (GSEA) on the up- and down-regulated DEGs was performed by “fgsea” package.

### Gene-miRNA interaction analysis, molecular docking, and network visualization

TargetScan (McGeary et al., 2019) was used to predict the interacting miRNAs of the genes in the genetic diagnostic signature and explore whether potential miRNAs are involved in the process of osteoporosis by targeting genes. Subsequently, miRPath was used for pathway enrichment analysis of miRNAs (Vlachos et al., 2012).

Firstly, we screened potential small molecule substances and drugs from the CTD database (Davis et al., 2021) and DGIdb database. Cytoscape was used to construct the miRNA-gene-molecule network. Secondly, we downloaded the protein 3D structures from the PDB database (Burley et al., 2021) and used PyMOL software to remove the water molecules of the protein. Then, we collected the 3D structures of these potential small-molecule substances and drugs from PubChem. Next, the SwissDock (Grosdidier et al., 2011) database was used for protein-molecule docking. Finally, PyMOL software was used to modify the docking results.

### RNA extraction and quantitative real-time polymerase chain reaction

Fourteen women with PMOP and 14 healthy postmenopausal-matched women controls were selected, and PBMCs were obtained from them. The characteristics of the patients are summarized in Table 1. This study was approved by the Ethics Committee of the First Affiliated Hospital of Jinan University. Both patients and controls provided written informed consent.

**TABLE 1:** Patient characteristics (*n* = 28).

	Mean ± SD (Range)	
Characteristic	High bone density	Low bone density
Patients (n)	14	14
Age (years)	61.9 ± 9.5	66.2 ± 8.4
Height (cm)	156.8 ± 6,7	158.6 ± 5.6
Weight (kg)	58.4 ± 6.3	61.1 ± 7.6
T-score	0.7 ± 0.9	-2.4 ± 0.8
Menstrual condition		
Menopause	14	14
Premenopausal	0	0
Smoking status		
Smoking	0	0
No history of smoking	14	14
Surgery situation		
Have vertebroplasty	0	0
No vertebroplasty	14	14

PBMCs were extracted with Histopaque-1077 (Sigma, United States). According to the manufacturer’s protocol, the total RNA of PBMCs from all samples was extracted using an EZ-Press RNA Purification Kit (EZbioscience, United States). cDNA was obtained by reverse transcription using a PrimeScript RT Kit (TaKaRa, Japan). Based on the SYBR Green method (ChamQ Universal SYBR qPCR Master Mix, Vazyme Biotech, China), The CFX96 Real-Time PCR System (Bio-Rad, United States) was used for RT-qPCR detection. The mRNA-specific primer sequences are shown in Table 2. After the expression level of GAPDH was used for normalization, the relative expression level of mRNA was determined.

**TABLE 2:** mRNA-specific primer sequences.

Gene	Primer sequence	Tm
*METTL4*	F: GCT​GTT​CAT​AAA​GAA​TGC​CAG​CAA	57
	R: CAG​CTC​CCT​GAT​CTT​TGT​ATG​GT	56
*RAB2A*	F: TCC​ATC​ACA​AGG​TCG​TAT​TAC​AGA	55
	R: TGG​TTG​AAT​GTA​TCT​CTC​CGT​GTA	55
*GAPDH*	F: ACAGTTGCCATGTAGACC	56
	R: TTTTTGGTTGAGCACAGG	60

### Statistical analysis

Statistical analyses were conducted using RStudio version 1.3.1093 (RStudio Inc.) and GraphPad Prism 8 (GraphPad Software, Inc.). All data are expressed as the mean ± SD. A paired difference test between Low BMD samples and normal control samples in two groups by the “limma” package was used to determine the DEGs. Analysis with one-way ANOVA followed by the Student–Newman–Keuls multiple comparison test was used for the comparison of three or more experimental groups. For qPCR data, Student’s t test was used for analysis.

## Results

### Quality control of microarray data and DEG screening

**FIGURE 1:**
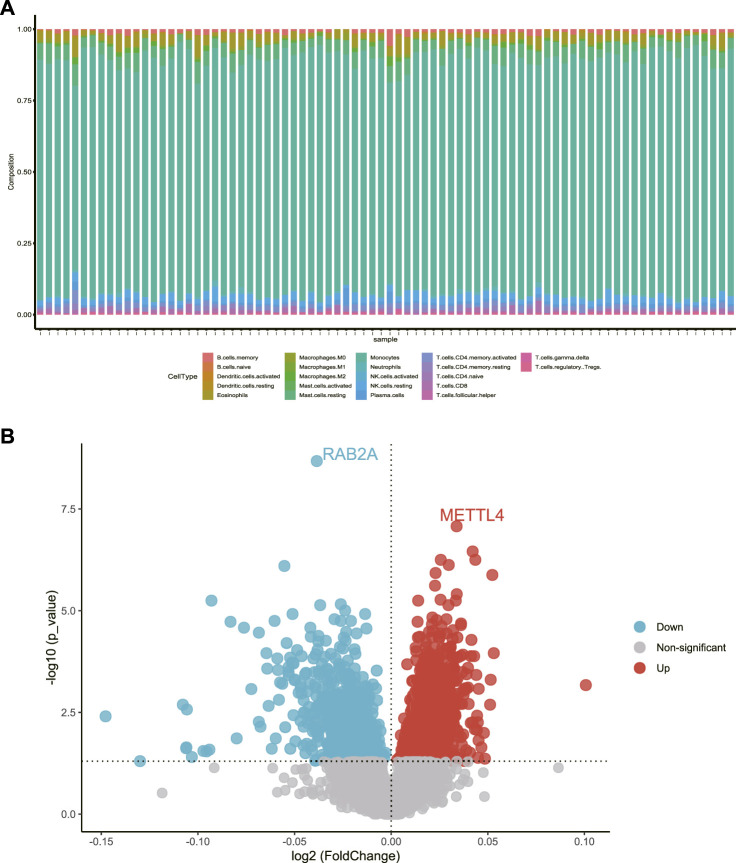
**(A)** Cell abundance of the pilot cohort. **(B)** Volcano plot of DEGs between the individuals with a high BMD and a low BMD in the pilot cohort.

First, cell purity analysis was performed on the pilot cohort (GSE56815), and the results are shown in Figure 1A, indicating that PBMCs accounted for the majority of the pilot cohort, consistent with the description in NCBI. DEG analysis was performed on the pilot cohort, and patients were divided into two groups according to BMD. DEGs were identified by “limma” package analysis, and 2,420 DEGs were screened out, as shown in Figure 1B.

### Weighted coexpression network and identification of bone mineral density-related modules

**FIGURE 2:**
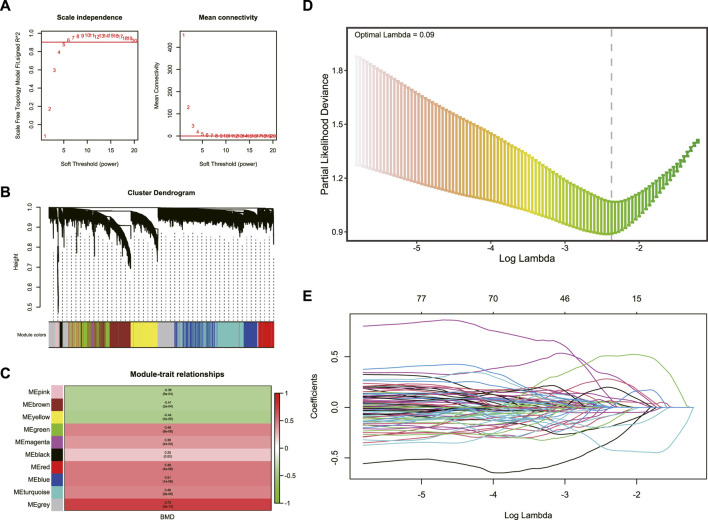
**(A)** Fitting index of the scale-free topology module under different soft thresholds (left) and network connectivity under different soft thresholds (right). **(B)** Cluster diagram of genes and the corresponding gene modules. **(C)** Correlation between module eigengenes and BMD. **(D)** Selection of the optimal parameter (λ) of LASSO regression through cross-validation. **(E)** LASSO coefficient profiles of the 15 genes that comprise the diagnostic signature selected by λ.

In this study, the “WGCNA” package was used to construct a weighted gene coexpression network in the pilot cohort. According to several iterations, *β* = 6 was selected as the optimal soft threshold to construct a scale-free network (Figure 2A). After exclusion of the MEgrey module, which contains all genes not involved in clustering, a total of 7 modules were identified. The interaction between modules was analyzed, and the heatmap showed that the gene expression of each module was relatively independent (Figure 2B). Then, correlation analysis between these modules and BMD was carried out, and the results showed that the MEblue module, which consisted of 396 genes, had the highest correlation with BMD (cor = 0.51, *p* < 0.001) (Figure 2C).

### Construction and testing of the diagnostic signature

**FIGURE 3:**
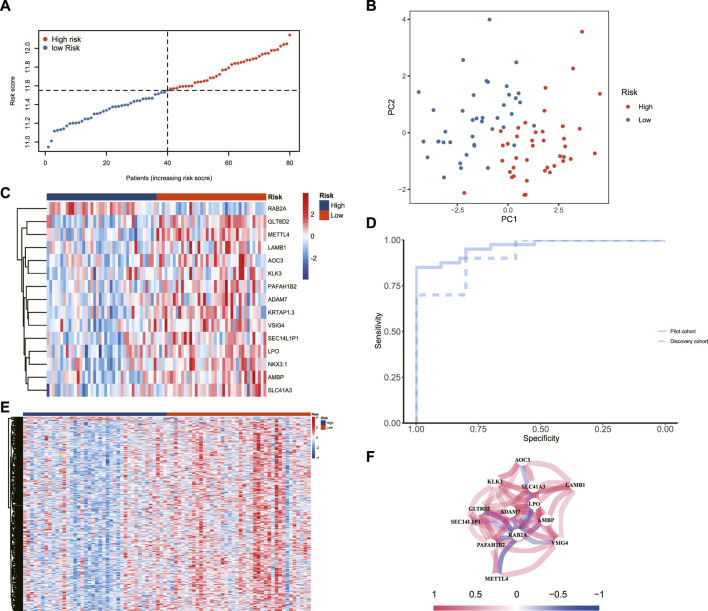
**(A)** Risk plot and **(B)** PCA of the pilot cohort. **(C)** The heatmap shows the different expression patterns of 15 genes. **(D)** ROC curves of the pilot cohort and the validation cohort. **(E)** The heatmap shows the different expression patterns between the high- and low-risk groups. **(F)** Correlations among the 15 genes.

The LASSO algorithm was used to determine *λ* = 0.09 (Figure 2D), and a diagnostic signature consisting of 15 genes (Figure 2E) was established. The specific gene composition and coefficient of each gene are shown in Table 3. The diagnostic signature calculated the patients’ risk scores and divided them into high-risk and low-risk groups (Figure 3A). Principal component analysis (PCA) showed that risk scores could categorize patients with different BMDs in the pilot cohort into two groups (Figure 3B). Moreover, verification was carried out in this study, and the results showed that the AUC value of the diagnostic signature was 0.993 in the pilot cohort and 0.980 in the discovery cohort (Figure 3C). The expression patterns of the 15 genes that constituted the signature (Figure 3D) and all the DEGs (Figure 3E) in the high-risk and low-risk groups are shown. Heatmap results showed that the gene expression patterns of the high-risk and low-risk groups were different, especially those of the 15 genes that constituted the signature. All the above results showed that this signature has an excellent ability to predict the risk of osteoporosis. In addition, we explored the interactions of these 15 genes (Figure 3F).

**TABLE 3:** Genes and their coefficients that constitute the diagnostic signature.

Gene	Coefficient
***RAB2A***	−0.69559
*VSIG4*	0.005073
*ADAM7*	0.014223
*AMBP*	0.018658
*PAFAH1B2*	0.031496
*AOC3*	0.050969
*KLK3*	0.088715
*KRTAP1.3*	0.100,972
*LPO*	0.13443
*SLC41A3*	0.144,549
*NKX3.1*	0.208,241
*GLT8D2*	0.231,434
*LAMB1*	0.338,636
*SEC14L1P1*	0.394,242
***METTL4***	1.089913

### Functional annotation and Gene Set Enrichment Analysis

**FIGURE 4:**
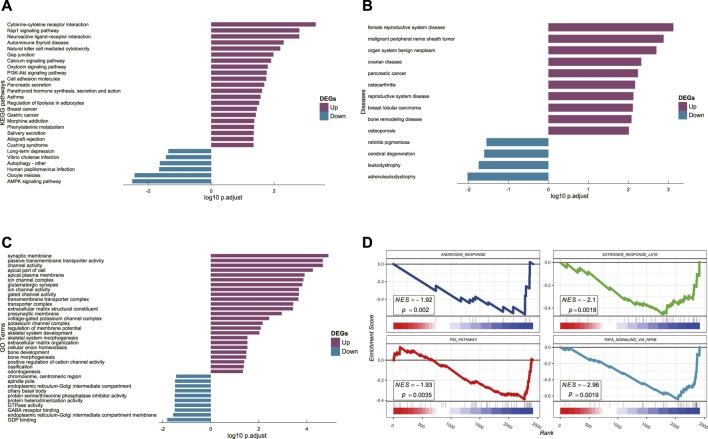
**(A)** Result of KEGG enrichment analysis. **(B)** Result of DO analysis. **(C)** Result of GO analysis. **(D)** Results of the GSEA.

KEGG functional annotation analysis (Figure 4A) showed that cytokine receptor interaction, neural activity ligand–receptor interaction, Rap1 signaling pathway, autoimmune thyroid disease, natural killer cell-mediated cytotoxicity, PI3K-Akt signaling pathway, gap junction, calcium signaling pathway, and oxytocin signaling pathway were up-regulated. Combining the above results with previous studies, we found that the calcium activator calcimycin can activate the RAF/MEK/ERK pathway through Ras (Li et al., 2005), and increased calcium concentrations have also been shown to modulate Ras-dependent Raf1 activation (Yoshiki et al., 2010). Moreover, lactoferrin-induced PI3K-Akt pathway activation and Ras phosphorylation can promote osteoblast proliferation (Hou et al., 2015). DO analysis (Figure 4B) showed that gynecological and aging diseases were up-regulated, such as female reproductive system disease, ovarian disease, bone remodeling disease, osteoporosis, and bone resorption diseases. GO analysis (Figure 4C) showed that ion channel complex activity, bone development, bone morphogenesis were up-regulated, and odontogenesis, GTPase activity, GDP binding were down-regulated.

According to the GSEA results (Figure 4D), we suggest that androgen response, late estrogen response, the P53 pathway, and TNF-α signaling *via* NF-κB are were down-regulated in the DEGs of osteoporosis.

### miRNA interaction identification and candidate molecule prediction

**FIGURE 5:**
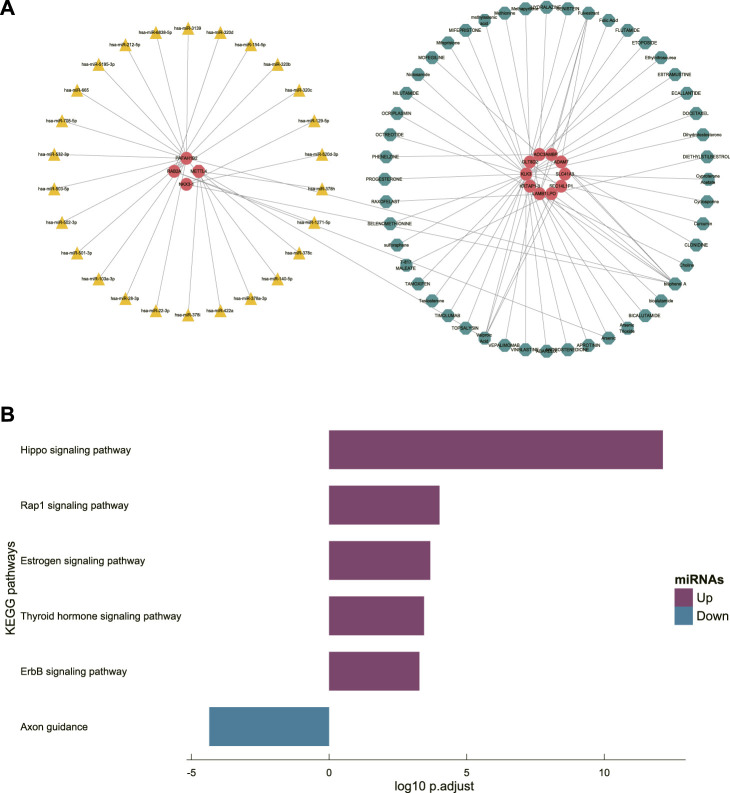
**(A)** miRNA-gene-molecule interaction network. **(B)** KEGG enrichment analysis of the potential interacting miRNAs.

Thirty-eight miRNAs were expected to interacted with the genes constituting the diagnostic signature and were used to construct the miRNA-Gene-Molecule network (Figure 5A). The result of KEGG (Figure 5B) enrichment analysis showed that miRNA pathway enrichment overlapped with DEG pathways, such as the Rap1 signaling pathway, suggesting that overlapping pathways play a potentially important role in the occurrence and development of PMOP. We selected the molecules connected to at least two genes as the candidate molecules. Five candidate molecules were screened out, including bisphenol A (BPA), fulvestrant, bicalutamide, mifepristone, and valproic acid (VPA). RAF, an essential protein in the Rap1 signaling pathway, was selected for docking with the candidate molecules to explore their possible binding locations (Supplementary Figure S1).

### Validation by real-time polymerase chain reaction

**FIGURE 6:**
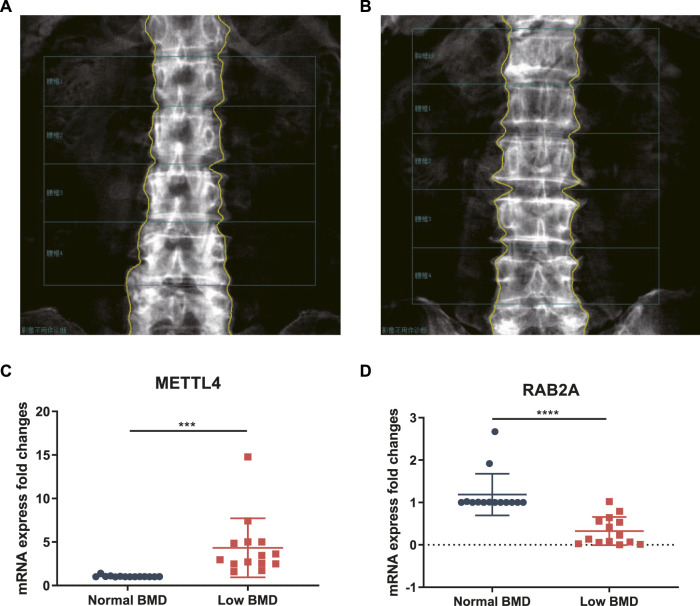
DXA images of the lumbar spine L1-L4 of women with a normal BMD **(A)** and low BMD **(B)**. The overall expression of *METTL4* **(C)**and *RAB2A* **(D)** in PBMCs from 14 low BMD patients and healthy controls.

To verify the authenticity of the diagnostic signature, we collected PBMCs from 14 postmenopausal healthy controls (Figure 6A) and 14 postmenopausal women with low BMD (Figure 6B) in this study. RNA was extracted for RT–qPCR to verify the diagnostic signature. The gene with the most significant coefficient had the strongest contribution to the risk score, and the gene with the largest AUC showed the strongest relationship to BMD. Interestingly, *METTL4* and *RAB2A* had the largest positive and negative coefficients, respectively, and they also had the most significant AUC values (Table 4). Therefore, these two genes were further investigated, and the results were consistent with the bioinformatics results. The expression of *METTL4* in the low BMD group was significantly higher than that in the normal BMD group (Figure 6C), while the expression of *RAB2A* in the normal BMD group was significantly higher than that in the low BMD group (Figure 6D), suggesting that these two genes play an essential role in the occurrence and development of osteoporosis.

**TABLE 4:** Genes and their AUCs that constitute the diagnostic signature.

Gene	AUC
***RAB2A***	0.8638
*VSIG4*	0.7431
*ADAM7*	0.7219
*AMBP*	0.72
*PAFAH1B2*	0.725
*AOC3*	0.6444
*KLK3*	0.6444
*KRTAP1.3*	0.7375
*LPO*	0.7141
*SLC41A3*	0.7338
*NKX3.1*	0.7075
*GLT8D2*	0.7819
*LAMB1*	0.6481
*SEC14L1P1*	0.7038
***METTL4***	0.8306

## Discussion

Osteoporosis is a systemic bone disease that mainly involves decreased bone mass and increased bone brittleness caused by degeneration of the bone tissue microstructure, resulting in susceptibility to fracture (Wang et al., 2020). DXA is widely used in clinical practice as a diagnostic standard for osteoporosis. However, only a small number of postmenopausal women are tested for BMD, and many of them have already suffered from brittle fractures when BMD is found to be reduced (Siris et al., 2014). BMD examination results may be affected by body weight, lumbar curvature, osteophytes, vertebral fractures, and vascular calcification. Studies have shown that vertebral osteoarthropathy and aortic calcification can cause false BMD increases and decrease diagnostic sensitivity (Orwoll et al., 1990; Frohn et al., 1991).

Relying solely on BMD testing will lead to the failure of clinicians and patients to correctly evaluate the severity of osteoporosis, thus affecting clinical treatment strategies. In addition, the radiation of DXA is very low, with each scan receiving only 1/60 to 1/10 of the radiation dose of a conventional X-ray. However, repeated exposure to ionizing radiation over a long period can have long-term health effects, including cancer (Hill and Einstein, 2016; Howard et al., 2020), suggesting that frequent imaging detection is still not advisable.

Due to the difficulty in the extraction and separation of osteoblasts and osteoclasts, on the contrary, the isolation and extraction technology of PBMCs has become increasingly mature, and its extraction and purification rate can reach more than 90%. PBMCs are the most likely precursors of osteoclasts, especially in adult peripheral bone, and are the only precursors of osteoclasts (Fujikawa et al., 1996). Secondly, PBMCs can secrete cytokines such as IL-1B, IL-6, and TNF-α, which play an essential role in osteoclast differentiation, activation, and apoptosis (Custer and Ahlfeldt, 1932). The decrease of PBMCs cytokines is the primary mechanism by which sex hormones inhibit osteoclast formation and bone resorption. Therefore, PBMCs are widely used as an ideal cell model for osteoporosis study (Zhou et al., 2015).

Previous studies have shown that osteoporosis is a polygenic disease, and genetic factors play an essential role in the occurrence and development of this condition. However, there are few relevant studies at present, and few candidate genes have been identified (Andrew and Macgregor, 2004; Cai et al., 2018). Thus, we used public databases to construct a transcriptome-based diagnostic signature, which is expected to be applied in clinical practice in the future to assess the risk of osteoporosis in the potential population and help doctors diagnose this disease. In this study, the GEO dataset GSE56815 was first used to identify DEGs between patients with osteoporosis and healthy controls, and then WGCNA was performed on these DEGs. The results showed that the blue module was most closely related to osteoporosis. Then, the genes in the blue module were extracted, and a diagnostic signature composed of 15 genes was constructed through LASSO regression. Subsequently, the independent dataset GSE13850 was used to verify the classification ability of the signature. We found that the AUC values of the signature in the pilot cohort and the discovery cohort were 0.993 and 0.920, respectively, showing good prediction of the risk of osteoporosis in different patients.

To further explore the potential pathogenesis of osteoporosis, we conducted the functional enrichment analysis of DEGs between the high- and low-BMD groups and interacting miRNAs of the diagnostic signature. We found that both were involved with the Rap1 signaling pathway, suggesting that, compared with other mechanisms, the Rap1 signaling pathway may be more closely related to our diagnostic signature. The calcium activator calcimycin can activate the RAF-MEK-ERK pathway through the RAS signaling pathway (Li et al., 2005). An increased calcium concentration has also been shown to modulate RAS-dependent RAF1 activation (Yoshiki et al., 2010), and lactoferrin-induced PI3K-Akt pathway activation and Ras phosphorylation can promote osteoblast proliferation (Hou et al., 2015). According to previous studies, osteoclasts are specialized macrophage/monocyte lineage-derived cells that resorb bone, and neurofibromatosis type I (NF1) haploinsufficient osteoclasts have abnormal Ras-dependent bone resorption. (Yan et al., 2008).

Our study found that the two genes with the most significant positive and negative coefficients in the diagnostic signature, ***METTL4*** and ***RAB2A***, were also the two genes with the most significant AUC values among the 15 genes. Therefore, we selected these two genes as representative molecules that were validated by RT–qPCR. We collected PBMCs from 14 patients with normal BMD and 14 patients with low BMD for RNA extraction. The results showed that ***METTL4*** expression was significantly lower in patients with normal BMD than in patients with low BMD, while ***RAB2A*** expression was significantly higher in healthy controls than in patients with low BMD, which was consistent with our bioinformatics results and the results of other studies. ***RAB2A*** is a member of the Ras gene family. When it binds to GTP, the Ras protein can phosphorylate and activate downstream proteins, thus regulating the proliferation and differentiation of osteoblasts (Ge et al., 2007). The Ras gene family regulates anterograde transport from the endoplasmic reticulum to the Golgi complex, inhibits the proliferation and differentiation of osteoprogenitor MC3T3-E1 cells and promotes apoptosis by reducing the membrane transport process. During this process, the protein expression of ***RAB2A*** was inhibited (Hong et al., 2011). METTL4, as an m6A methyltransferase, can lead to increased m6A modification. As a new extranuclear marker, m6A modification is involved in bone development and metabolism and plays an essential role in osteoporosis. A previous study showed that both m6A levels and methyltransferase expression are increased during osteoclast differentiation, and methyltransferase knockdown led to increased osteoclast volume and decreased bone resorption capacity (Li et al., 2020). Another study also showed that inhibition of mRNA methyltransferase reversed osteoclast differentiation and bone resorption (Wang et al., 2021).

Another vital function of diagnostic signatures is to provide evidence for candidate drugs. RAF is a crucial component of the Rap1 signaling pathway, and RAF has also been shown to affect the proliferation and function of osteoblasts (Meng et al., 2015). Therefore, it is necessary to develop effective new osteoporosis drugs that target RAF. This study screened out five medications with high affinity for the diagnostic signature: BPA, fulvestrant, mifepristone, bicalutamide, and VPA. Although the specific mechanisms of action of these small compounds remain to be further elucidated, our results suggest that they have therapeutic potential for osteoporosis, especially in patients with PMOP.

We speculated that using transcriptome analysis to detect gene expression might be a complementary method for the diagnosis of osteoporosis, and the genes can be used as biomarkers to evaluate the effect of osteoporosis treatment to avoid frequent radiation examinations in patients.

## Data Availability

The original contributions presented in the study are included in the article/supplementary materials, further inquiries can be directed to the corresponding author.

## References

[B1] Andrew T. Macgregor A. J. (2004). Genes and osteoporosis. Curr. Osteoporos. Rep. 2 (3), 79–89. Epub 2005/07/23. 10.1007/s11914-004-0015-1 16036087

[B2] Barrett T. Wilhite S. E. Ledoux P. Evangelista C. Kim I. F. Tomashevsky M. (2013). Ncbi geo: Archive for functional genomics data sets--update. Nucleic Acids Res. 41, D991–D995. Database issueEpub 2012/11/30. 10.1093/nar/gks1193 23193258 PMC3531084

[B3] Burley S. K. Bhikadiya C. Bi C. Bittrich S. Chen L. Crichlow G. V. (2021). Rcsb protein data bank: Powerful new tools for exploring 3d structures of biological macromolecules for basic and applied research and education in fundamental biology, biomedicine, Biotechnology, bioengineering and energy sciences. Nucleic Acids Res. 49 (D1), D437–D451. Epub 2020/11/20. 10.1093/nar/gkaa1038 33211854 PMC7779003

[B4] Cai X. Yi X. Zhang Y. Zhang D. Zhi L. Liu H. (2018). Genetic susceptibility of postmenopausal osteoporosis on sulfide quinone reductase-like gene. Osteoporos. Int. 29 (9), 2041–2047. Epub 2018/06/02. 10.1007/s00198-018-4575-9 29855663

[B5] Cheng X. Zhao K. Zha X. Du X. Li Y. Chen S. (2021). Opportunistic screening using low-dose ct and the prevalence of osteoporosis in China: A nationwide, multicenter study. J. Bone Min. Res. 36 (3), 427–435. Epub 2020/11/05. 10.1002/jbmr.4187 PMC7988599 33145809

[B6] Custer R. Ahlfeldt F. E. (1932). Studies on the structure and function of bone marrow: Ii. Variations in cellularity in various bones with advancing years of life and their relative response to stimuli. J. Laboratory Clin. Med. 17 (10), 960–962.

[B7] Davis A. P. Grondin C. J. Johnson R. J. Sciaky D. Wiegers J. Wiegers T. C. (2021). Comparative toxicogenomics database (ctd): Update 2021. Nucleic Acids Res. 49 (D1), D1138–D1143. Epub 2020/10/18. 10.1093/nar/gkaa891 33068428 PMC7779006

[B8] Ensrud K. E. Kats A. M. Boyd C. M. Diem S. J. Schousboe J. T. Taylor B. C. (2019). Association of disease definition, comorbidity burden, and prognosis with hip fracture probability among late-life women. JAMA Intern. Med. 179 (8), 1095–1103. Epub 2019/06/18. 10.1001/jamainternmed.2019.0682 31206140 PMC6580441

[B9] Friedman J. Hastie T. Tibshirani R. (2010). Regularization paths for generalized linear models via coordinate descent. J. Stat. Softw. 33 (1), 1–22. Epub 2010/09/03. 10.18637/jss.v033.i01 20808728 PMC2929880

[B10] Frohn J. Wilken T. Falk S. Stutte H. J. Kollath J. Hör G. (1991). Effect of aortic sclerosis on bone mineral measurements by dual-photon absorptiometry. J. Nucl. Med. 32 (2), 259–262. Epub 1991/02/01. 1992030

[B11] Fujikawa Y. Quinn J. M. Sabokbar A. McGee J. O. Athanasou N. A. (1996). The human osteoclast precursor circulates in the monocyte fraction. Endocrinology 137 (9), 4058–4060. Epub 1996/09/01. 10.1210/endo.137.9.8756585 8756585

[B12] Gautier L. Cope L. Bolstad B. M. Irizarry R. A. (2004). Affy--Analysis of Affymetrix genechip data at the probe level. Bioinformatics 20 (3), 307–315. Epub 2004/02/13. 10.1093/bioinformatics/btg405 14960456

[B13] Ge C. Xiao G. Jiang D. Franceschi R. T. (2007). Critical role of the extracellular signal-regulated kinase-mapk pathway in osteoblast differentiation and skeletal development. J. Cell Biol. 176 (5), 709–718. Epub 2007/02/28. 10.1083/jcb.200610046 17325210 PMC2064027

[B14] Glaser D. L. Kaplan F. S. (1976). Osteoporosis. Definition and clinical presentation. Spine 22 (24), 12S–16S. Epub 1998/02/07. 10.1097/00007632-199712151-00003 9431639

[B15] Grosdidier A. Zoete V. Michielin O. (2011). Swissdock, a protein-small molecule docking web service based on eadock dss. Nucleic Acids Res. 39, W270–W277. Web Server issue)Epub 2011/06/01. 10.1093/nar/gkr366 21624888 PMC3125772

[B16] Hill K. D. Einstein A. J. (2016). New approaches to reduce radiation exposure. Trends cardiovasc. Med. 26 (1), 55–65. Epub 2015/05/13. 10.1016/j.tcm.2015.04.005 25962784 PMC4607546

[B17] Hong D. Chen H. X. Yu H. Q. Wang C. Deng H. T. Lian Q. Q. (2011). Quantitative proteomic analysis of dexamethasone-induced effects on osteoblast differentiation, proliferation, and apoptosis in mc3t3-E1 cells using silac. Osteoporos. Int. 22 (7), 2175–2186. Epub 2010/11/10. 10.1007/s00198-010-1434-8 21060993 PMC4507272

[B18] Hou J. M. Chen E. Y. Lin F. Lin Q. M. Xue Y. Lan X. H. (2015). Lactoferrin induces osteoblast growth through igf-1r. Int. J. Endocrinol. 2015, 282806. Epub 2015/08/21. 10.1155/2015/282806 26290662 PMC4531176

[B19] Howard A. West R. M. Iball G. Panteli M. Baskshi M. S. Pandit H. (2020). Should radiation exposure Be an issue of concern in children with multiple trauma? Ann. Surg. 275, 596–601. Epub 2020/08/03. 10.1097/sla.0000000000004204 32740254

[B20] Ito K. Murphy D. (2013). Application of Ggplot2 to pharmacometric graphics. CPT. Pharmacometrics Syst. Pharmacol. 2 (10), e79. Epub 2013/10/18. 10.1038/psp.2013.56 24132163 PMC3817376

[B21] Langfelder P. Horvath S. (2008). Wgcna: An R package for weighted correlation network analysis. BMC Bioinforma. 9, 559. Epub 2008/12/31. 10.1186/1471-2105-9-559 PMC2631488 19114008

[B22] Li D. Cai L. Meng R. Feng Z. Xu Q. (2020). Mettl3 modulates osteoclast differentiation and function by controlling rna stability and nuclear export. Int. J. Mol. Sci. 21 (5), E1660. Epub 2020/03/04. 10.3390/ijms21051660 32121289 PMC7084668

[B23] Li D. W. Liu J. P. Mao Y. W. Xiang H. Wang J. Ma W. Y. (2005). Calcium-activated raf/mek/erk signaling pathway mediates P53-dependent apoptosis and is abrogated by alpha B-crystallin through inhibition of Ras activation. Mol. Biol. Cell 16 (9), 4437–4453. Epub 2005/07/08. 10.1091/mbc.e05-01-0010 16000378 PMC1196350

[B24] McGeary S. E. Lin K. S. Shi C. Y. Pham T. M. Bisaria N. Kelley G. M. (2019). The biochemical basis of microrna targeting efficacy. Science 366 (6472), eaav1741. Epub 2019/12/07. 10.1126/science.aav1741 31806698 PMC7051167

[B25] Meng H. Z. Zhang W. L. Liu F. Yang M. W. (2015). Advanced glycation end products affect osteoblast proliferation and function by modulating autophagy via the receptor of advanced glycation end products/raf protein/mitogen-activated protein kinase/extracellular signal-regulated kinase kinase/extracellular signal-regulated kinase (Rage/Raf/Mek/Erk) pathway. J. Biol. Chem. 290 (47), 28189–28199. Epub 2015/10/17. 10.1074/jbc.M115.669499 26472922 PMC4653677

[B26] Orwoll E. S. Oviatt S. K. Mann T. (1990). The impact of osteophytic and vascular calcifications on vertebral mineral density measurements in men. J. Clin. Endocrinol. Metab. 70 (4), 1202–1207. Epub 1990/04/01. 10.1210/jcem-70-4-1202 2318940

[B27] Ritchie M. E. Phipson B. Wu D. Hu Y. Law C. W. Shi W. (2015). Limma powers differential expression analyses for rna-sequencing and microarray studies. Nucleic Acids Res. 43 (7), e47. Epub 2015/01/22. 10.1093/nar/gkv007 25605792 PMC4402510

[B28] Robin X. Turck N. Hainard A. Tiberti N. Lisacek F. Sanchez J. C. (2011). Proc: An open-source package for R and S+ to analyze and compare roc curves. BMC Bioinforma. 12, 77. Epub 2011/03/19. 10.1186/1471-2105-12-77 PMC3068975 21414208

[B29] Siris E. S. Adler R. Bilezikian J. Bolognese M. Dawson-Hughes B. Favus M. J. (2014). The clinical diagnosis of osteoporosis: A position statement from the national bone health alliance working group. Osteoporos. Int. 25 (5), 1439–1443. Epub 2014/03/01. 10.1007/s00198-014-2655-z 24577348 PMC3988515

[B30] Vlachos I. S. Kostoulas N. Vergoulis T. Georgakilas G. Reczko M. Maragkakis M. (2012). Diana mirpath V.2.0: Investigating the combinatorial effect of micrornas in pathways. Nucleic Acids Res. 40, W498–W504. Web Server issueEpub 2012/06/01. 10.1093/nar/gks494 22649059 PMC3394305

[B31] Wang H. Zhao W. Tian Q. J. Xin L. Cui M. Li Y. K. (2020). Effect of lncrna Ak023948 on rats with postmenopausal osteoporosis via pi3k/akt signaling pathway. Eur. Rev. Med. Pharmacol. Sci. 24 (5), 2181–2188. Epub 2020/03/21. 10.26355/eurrev_202003_20483 32196569

[B32] Wang W. Qiao S. C. Wu X. B. Sun B. Yang J. G. Li X. (2021). Circ_0008542 in osteoblast exosomes promotes osteoclast-induced bone resorption through M6a methylation. Cell Death Dis. 12 (7), 628. Epub 2021/06/20. 10.1038/s41419-021-03915-1 34145224 PMC8213782

[B33] Yan J. Chen S. Zhang Y. Li X. Li Y. Wu X. (2008). Rac1 mediates the osteoclast gains-in-function induced by haploinsufficiency of Nf1. Hum. Mol. Genet. 17 (7), 936–948. Epub 2007/12/20. 10.1093/hmg/ddm366 18089636

[B34] Yao P. Bennett D. Mafham M. Lin X. Chen Z. Armitage J. (2019). Vitamin D and calcium for the prevention of fracture: A systematic review and meta-analysis. JAMA Netw. Open 2 (12), e1917789. Epub 2019/12/21. 10.1001/jamanetworkopen.2019.17789 31860103 PMC6991219

[B35] Yoshiki S. Matsunaga-Udagawa R. Aoki K. Kamioka Y. Kiyokawa E. Matsuda M. (2010). Ras and calcium signaling pathways converge at Raf1 via the Shoc2 scaffold protein. Mol. Biol. Cell 21 (6), 1088–1096. Epub 2010/01/15. 10.1091/mbc.e09-06-0455 20071468 PMC2836960

[B36] Yu G. Wang L. G. Han Y. He Q. Y. (2012). Clusterprofiler: An R package for comparing biological themes among gene clusters. Omics 16 (5), 284–287. Epub 2012/03/30. 10.1089/omi.2011.0118 22455463 PMC3339379

[B37] Zhou Y. Deng H. W. Shen H. (2015). Circulating monocytes: An appropriate model for bone-related study. Osteoporos. Int. 26 (11), 2561–2572. Epub 2015/07/22. 10.1007/s00198-015-3250-7 26194495

