# Supplementary material for: Identification of a potential diagnostic signature for postmenopausal osteoporosis via transcriptome analysis
Source: Front Pharmacol. 2022 Aug 29;13:944735. doi: 10.3389/fphar.2022.944735 (PMC9464864; doi:10.3389/fphar.2022.944735)
Supplement: Supplementary file 1 [file DataSheet1.docx]

**Supplementary figure legend**

**Figure S1** 3D molecular-protein docking map. (**A**) Bisphenol A, (**B**) valproic acid, (**C**) bicalutamide, (**D**) fulvestrant, and (**E**) mifepristone docking with RAF.
