# Supplementary figures and images for: Identification of a potential diagnostic signature for postmenopausal osteoporosis via transcriptome analysis
Source: Front Pharmacol. 2022 Aug 29;13:944735. doi: 10.3389/fphar.2022.944735 (PMC9464864; doi:10.3389/fphar.2022.944735)

**A**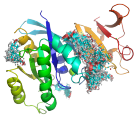**B**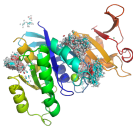**C**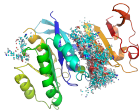**D**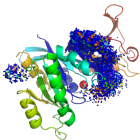**E**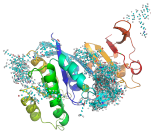

Supplement: Supplementary file 2 [file Image1.PDF]
